# Patient management pathways in dementia – Resource utilisation, diagnosis and drug treatment in the Stockholm region, Sweden

**DOI:** 10.1016/j.tjpad.2025.100132

**Published:** 2025-03-17

**Authors:** Emil Aho, Dorota Religa, Mozhu Ding, Bengt Winblad, Linus Jönsson, Karin Modig

**Affiliations:** a Division of Neurogeriatrics, Department of Neurobiology, Care Sciences and Society, The Center for Alzheimer Research Karolinska Institutet, Stockholm, Sweden; b Division of Clinical geriatrics, Department of Neurobiology, Care Sciences and Society, The Center for Alzheimer Research Karolinska Institutet, Stockholm Sweden; c Unit of Epidemiology, Institute of Environmental Medicine, Karolinska Institutet, Stockholm, Sweden; d Theme Inflammation and Aging, Karolinska Univ Hospital, Huddinge, Sweden

**Keywords:** Dementia, Delivery of health care, Primary health care, Care management pathways, Anti-dementia drug treatment

## Abstract

**Background:**

New diagnostic and therapeutic options for Alzheimer's disease are beginning to be introduced and expected igto become more widely available in the coming years. Improved understanding of current pathways in diagnosis and initial care of patients with dementia can help inform choices around how best to integrate new technologies in existing care structures.

**Objectives:**

The aim of this study is to describe the care management pathways defined by the involvement of specialist and primary care for individuals with newly diagnosed dementia. It also seeks to characterise individuals in different management pathways based on resource use prior to diagnosis, the type of dementia diagnosis received, and the proportion who receive symptomatic anti-dementia drug treatment.

**Design:**

Observational cohort study

**Setting:**

Stockholm region, Sweden.

**Participants:**

All newly diagnosed dementia cases between 1st January 2018 to 30th June 2020 (*n* = 9,781). Dementia diagnoses in primary care were based on Regional Stockholm health care database and diagnoses in specialist care were based on the National Patient Register in Sweden.

**Measurements:**

Care management pathways were categorized into three groups: **primary care only** (diagnosed and followed up in primary care), **specialist, no follow-up** (diagnosed in specialist care but not followed up in specialist care), and **specialist with follow-up** (diagnosed and followed up in specialist care). These classifications were based on patients’ care episodes from the date of diagnosis and the subsequent 18 months. age at diagnosis, resource utilisation one-year prior diagnosis and diagnosis given and symptomatic anti-dementia treatment 18 months after initial diagnosis.

**Results:**

A total of 9,781 newly diagnosed dementia cases were identified. In the 18 months following diagnosis, 63 % of patients were diagnosed either partly or fully in specialist care, while 37 % were diagnosed solely in primary care. Patients diagnosed and managed only in primary care were older, spent more days in hospital, and received more social care in the year preceding their diagnosis. Their total care costs were also the highest. Alzheimer's disease was the most common diagnosis (48 %), while 27 % had an unspecified dementia diagnosis, varying by care setting (61 % for patients managed in primary care only and 6 % for patients diagnosed and followed up in specialist care). Overall, 47 % of patients received symptomatic anti-dementia treatment, with the highest share for patients diagnosed and followed up in specialist care (73 %) and the lowest in primary care only (19 %). Diagnosis varied by age and care setting Alzheimer's was most common in settings involving specialist care, whereas unspecified dementia was more common in primary care only regardless of age.

**Conclusion:**

The findings that patients managed exclusively in primary care were older, had higher pre-diagnosis resource utilisation, and were less likely to receive specific diagnoses or anti-dementia treatments highlight the crucial role of primary care in diagnosing and managing dementia among older individuals with complex needs. Further research is needed to explore primary care's role in diagnosis and treatment across diverse healthcare systems.

Future research is needed to explore whether and how new diagnostic tools and treatment for AD could facilitate timely diagnosis and care for older individuals with dementia in primary care.

## 1 Introduction

Major neurocognitive disorders, also referred to as dementia disorders, are estimated to affect over 55 million people worldwide and is estimated to cost society annually $ 1.3 trillion US dollars [1]. In Europe the number of patients with dementia is expected to increase by 60 % between 2025 and 2050 [2]. The costs associated with dementia increase with severity, and the annual cost per patient in severe stages are estimated to be about 590,000 – 800,000 SEK ([3,4]). (1 SEK ≈ 0.09 EUR or 0.1 USD). Early and accurate diagnosis is essential not only for initiating appropriate treatment but also for potentially mitigating the progression of the disease and its associated costs. Diagnosis of dementia involves a multi-step process. First, it is recommended to begin with a basic dementia assessment in primary care [5]. Referral to specialist care for extended assessment is recommended in cases where a basic dementia assessment is not sufficient for setting diagnosis or when there are other complicating factors, such as younger patients or conflicting test results in the basic assessment [5]. However, the level of adherence to this recommended diagnostic pathway is unknown and there is limited evidence on which patients get referred where and why. Timely diagnosis of cognitive impairment is important and enables the patients’ collaboration in the development of care plans, reduces disease burden through symptomatic treatment or lifestyle changes [[6], [7], [8]] and potentially reduces the societal cost of disease management [[9], [10], [11]]. Specific drug treatment for dementia disorders includes the class of acetylcholinesterase inhibitors (AChEI) and the NMDA receptor antagonist memantine, all indicated for dementia in Alzheimer's disease. AChEI are recommended for patients with mild to moderate AD dementia, while memantine for patients with moderate to severe AD dementia [12] but it is used through all dementia stages [13]. Both AChEIs and memantine are available as generics at a low cost. New diagnostic and therapeutic options primarily for Alzheimer's disease are beginning to be introduced and are expected to become more widely available in the coming years ([14,15]). Improved understanding of current pathways in diagnosis and initial care of patients with dementia can help inform choices around how best to integrate new technologies in existing care structures.

Region Stockholm is the capital region of Sweden and is responsible for providing health care to all 2.4 million inhabitants. Almost all health care given in the Stockholm Region is provided through publicly funded care, by a mix of public and private care providers.

Primary care plays an important role in initial diagnostics, coordinating the treatment and deciding which patients to refer to specialist care. Yet, the role of primary care in dementia treatment is understudied. One reason could be a lack of data. While the parts of the diagnostic process of dementia are studied, the management of patients is a lengthy process [16]. A better understanding of the flows of patients and the initial care burden of patients could help to inform the choices made, especially when new diagnostic methods and treatments become available.

This study aims to analyse the care management pathway of newly diagnosed dementia patients, to describe which individuals that are diagnosed and followed in primary or specialist care and how the diagnosis and treatment differ between these pathways. Objectives include describing the proportion of patients diagnosed and followed up in each care management pathway, patient characteristics, initial care burden at diagnosis as indicated by resource utilisation and overall costs prior to diagnosis, the specific diagnoses given, and the proportion receiving AChEIs or memantine across different management pathways and age groups.

## 2 Method

This observational cohort study utilised retrospectively collected administrative healthcare records on primary and specialist care, as well as prescribed medications. Data were obtained from Region Stockholm's administrative data warehouse, the National Patient Registry [17], and the National Prescribed Drug Registry [18] in Sweden to gather information on diagnoses, anti-dementia drug treatment and resource utilisation. Additionally, data on resource utilisation from social support services were retrieved from the National Registry of Care and Social Services for the Elderly and Persons with Impairments (2017–2021). The Register covers all individuals who receive municipal care under the Social Services Act and includes institutionalisation status, number of home care hours received, number of days receiving support for daytime activities, short-term care, and housing support. Details regarding death and place of residence were collected from the Total Population Registry [19]. The unique Swedish personal identification number was used to link data across these registries. The linked database provides comprehensive information about care visits, diagnoses, prescribed drug treatments and resource utilisation in primary, specialist, inpatient care, and social support for all individuals residing in the Stockholm region.

### 2.1 Study population

The study population included all individuals aged 60 years or older living in the Stockholm region who received a first-time dementia diagnosis between January 2018 and June 2020. Historical records about diagnoses before 2018 was used to ensure that no previous dementia diagnosis had been recorded (2002 – 2017 in the National Patient Registry and 2013 – 2017 in the Region Stockholm's administrative data warehouse). Patients were identified through a diagnosis of dementia from ICD-codes (ICD-10 codes; F00: Dementia in Alzheimer's disease, F01: Vascular dementia, F02: Dementia in other diseases classified elsewhere, F03: Unspecified dementia. F05.1: Delirium superimposed on dementia or G30: Alzheimer's disease). The index date was the diagnosis date.

Individuals were followed for 18 months, or until death or emigration from the time of their initial dementia diagnosis to analyse the care pathway, any changes in diagnosis, and the initiation of anti-dementia drug treatment.

### 2.2 Definitions

All diagnoses from a care visit were utilised to define dementia, regardless of whether they served as the primary or secondary diagnosis. Classification into etiological diagnoses was based on diagnoses from routine care, recorded according to International Classification of Disorders, 10th revision (ICD-10) [5]. Dementia diagnoses were hierarchically classified based on the most specific diagnosis as followed 1) Other specified dementia (F02, i.e. Dementia in Pick disease, Dementia in Parkinson's disease and Lewy body dementia), 2) Vascular dementia (F01), 3) Alzheimer's (F00 or G30), and 4) Unspecified dementia (F03 or F05.1). To ensure the highest diagnostic specificity, patients with multiple dementia diagnoses were categorised based on the most specific diagnosis. For example, if a patient had both "Other specified dementia" and "Alzheimer's disease," they were classified under "Other specified dementia."

Care management pathways were determined based on the setting of the dementia diagnosis. Patients were classified into primary care only or specialist care, depending on whether they had a recorded diagnosis in specialist care within the first 18 months after their initial dementia diagnosis. Patients with a specialist care diagnosis were further categorised as “specialist, with follow-up” if they had at least one follow-up visit in specialist care after the first 9 months but within the first 18 months of diagnosis or “specialist, no follow-up” if they had no follow-up visits in specialist care after the first 9 months within the first 18 months of diagnosis. The 9 months cut off was based on clinical information that follow-up is normally annual and we wanted to include a margin around that.

Specialist care consists of care providers in the medical fields of geriatrics or general psychiatric care for adults (medical fields that include all memory clinics in the Stockholm region). To ensure greater validity of the diagnosis recorded, only diagnoses provided by a physician are used and not visits that only included other profession for example nurses or physiotherapists. Anti-dementia treatment consists of AChEIs (ATC-code N06D) and memantine (ATC-code N06DX01). Social care incudes home help services, daytime activities, short-term care, and institutionalisation.

### 2.3 Statistical analysis

A descriptive methodology was used, Baseline characteristics of included patients were stratified by the whole cohort and care management pathway and were described by means and standard deviation for continuous outcomes, percentages for categorical variables and 95 % confidence intervals were calculated. Resource utilisation was calculated one year before first diagnosis All resources provided to the patient were included, not just those attributable to dementia. Costs for medical visits in specialist and hospital care were obtained by multiplying disease-related group (DRG) diagnoses in the National Patient Registry by year-specific DRG costs and weights. The cost for primary care was calculated in Swedish kronor (SEK; 1 SEK ≈ 0.09 EUR or 0.1 USD) by multiplying the number of visits recorded in the Region Stockholm's administrative data warehouse by an average cost per visit to a physician in primary care retrieved from Kostnad per patient (KPP), a database that provides average patient costs on care carried out by regions [20]. The cost for social care was calculated by multiplying the resource utilisation recorded in the National Registry of Care and Social Services for the Elderly and Persons with Impairments with respective unit costs retrieved from Kolada, a database that provides information on activities carried out by municipalities and regions [21]. All costs are calculated at 2022 price level. The unit costs are presented in Table 1 **in the supplementary material**. To calculate shares, the number of individuals with a certain diagnosis or outcome was divided by the total number of individuals in the stratum. A 95 % confidence interval was calculated using the Wilson formula. To test if there were differences between groups a one-way analysis of variance (ANOVA) test was used for continuous variables and the Kruskal-Wallis test for ordinal variables. R (version 4.3.3) in RStudio was used for statistical analysis [22].

**Table 1:** Number with newly diagnosed dementia cases between 1st of January 2018 – and 30th of June 2020, and resource utilisation one year prior diagnosis, stratified by management pathway.

	Care management pathway				
	All	Primary care only	Specialist, no follow-up	Specialist, with follow-up	p-value
n	9784	2492	4671	2621	
Age, mean (SD)	81.2 (7.8)	83.9 (8)	81.4 (7.4)	78.1 (7.3)	<0.01
Male %	40.2	36.8	40.0	43.8	<0.01
Resource utilisation, mean (SD)					
Primary care visits	5.5 (7.4)	5.0 (7.6)	5.8 (7.9)	5.6 (6)	<0.01
Outpatient specialist care visits	4.8 (6.7)	3.6 (7.3)	4.9 (6.8)	5.5 (5.7)	<0.01
Hospital days	6.8 (14)	8.3 (15.7)	7.4 (14.3)	4.5 (11.2)	<0.01
Home help services (hours)	179.9 (372.5)	216.7 (468.7)	206.2 (370.1)	97.9 (236.2)	<0.01
Daytime activities (months)	0.3 (1.6)	0.4 (1.8)	0.4 (1.7)	0.2 (1.2)	<0.01
Short term care (days)	2.4 (14.8)	4.2 (18.9)	2.4 (14.2)	0.8 (10.6)	<0.01
Institutionalisation (months)	1.1 (3.3)	3.3 (5)	0.5 (2.2)	0.1 (0.7)	<0.01
Cost thousand SEK, mean (SD)					
Primary care (cost)	10.1 (13.5)	9.1 (13.9)	10.7 (14.5)	10.2 (11)	<0.01
Outpatient specialist care (cost)	22.4 (31)	15.8 (34.1)	23.3 (31.5)	27.1 (25.3)	<0.01
Hospital (cost)	74.6 (143.4)	88.1 (160.9)	79.8 (145.2)	52.5 (117.9)	<0.01
Home help services (cost)	102.7 (212.7)	123.8 (267.6)	117.7 (211.3)	55.9 (134.9)	<0.01
Daytime activities (cost)	3.3 (15.9)	3.6 (17.3)	3.8 (16.9)	2.0 (12.1)	<0.01
Short term care (cost)	7.8 (47.8)	13.4 (61.1)	7.8 (45.9)	2.7 (34.2)	<0.01
Institutionalisation (cost)	100.8 (294.6)	300.7 (453.3)	47.6 (201.1)	5.4 (66.8)	<0.01
Total cost	321.8 (397.7)	554.4 (494)	290.7 (355.4)	155.9 (229.8)	<0.01

**Notes:** Primary care only, a patient only has recorded dementia diagnoses in primary care; Specialist, no follow-up, a patient has a recorded dementia diagnosis in specialist care, but was not followed up in specialist care; Specialist, with follow-up, a patient has a recorded dementia diagnosis in specialist care and was followed up in specialist care**;** the p-value states if there are significant differences between primary care only, specialist, no follow-up and specialist, with follow-up **.**

**Abbreviations:** SD, standard deviation; AChEI, acetylcholinesterase inhibitors.

## 3 Results

A total of 9781 newly diagnosed dementia cases were identified during the inclusion period from January 2018 to June 2020. In the first 18 months following the initial diagnosis, 75 % of patients were diagnosed and followed up partly or fully in specialist care. The mean age at diagnosis for the entire population was 81.2 years, and 40 % of the patients were male. There were statistically significant differences (*p* < 0,01) in the management pathways and resource utilisation by age and sex. Individuals diagnosed and followed up in primary care only were older (mean 83.9 years compared to 81.1 years in total population), to a larger share female, had more hospital days and received more social care (home help services, daytime activities, short-term care, and institutionalisation) one year prior to their diagnosis (Table 1).

All differences in resource utilisation were statistically significant (*p* < 0.01 for all). The average care cost per patients for the whole population was 321,800 SEK one year prior to diagnosis. Costs differed between care pathways (*p* < 0.01), where costs before diagnosis were highest for patients diagnosed and followed up in primary care only (555,200 SEK) and lowest for patients diagnosed and followed in specialist care (156,000 SEK). This were primarily due to differences in institutionalisation costs, which accounted for 54 % of total costs for patients in the primary care only group, while the share is 3 % in the specialist, with follow-up group. Patients in the primary care group also had higher costs for home help services and hospitalisation compared to other groups. Individuals diagnosed and followed up in specialist care had more visits in outpatient specialist care before diagnosis, 5.5 visits in specialist, with follow-up and 3.6 visits in primary care only (Table 1).

**Fig. 1:**
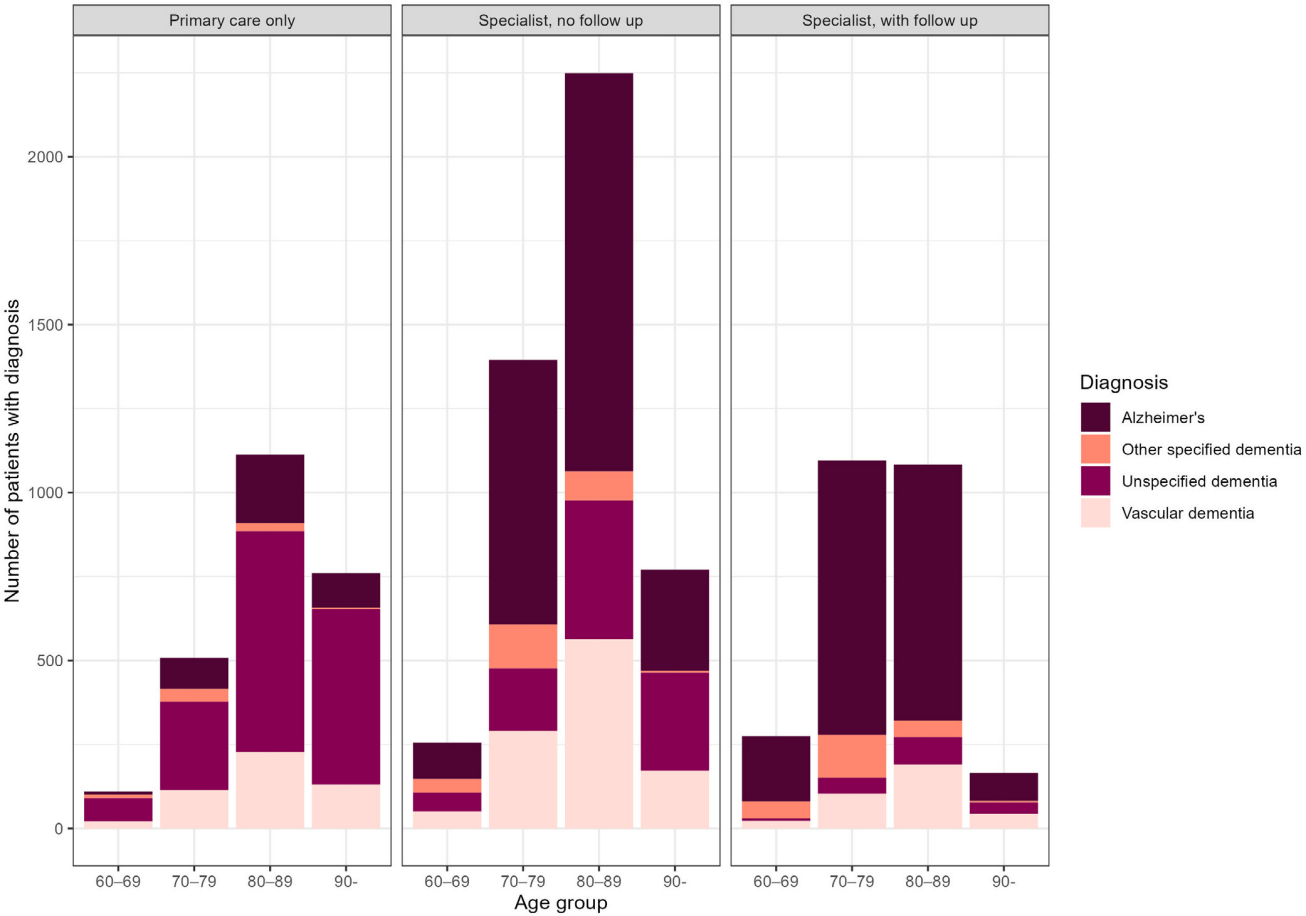
Diagnosis given within 18 months to newly diagnosed patients with dementia, 1st of January 2018 – and 30th of June 2020, stratified by management pathway. and age group. Notes: Primary care only, a patient only has recorded dementia diagnoses in primary care; Specialist, no follow-up, a patient has a recorded dementia diagnosis in specialist care but was not followed up in specialist care; Specialist, with follow-up, a patient has a recorded dementia diagnosis in specialist care and was followed up in specialist care.

The specific diagnose given differed between groups (*p* < 0.0.) Alzheimer's disease was the most common diagnosis, given to 48 % of all newly diagnosed individuals. Overall, 27 % were given an unspecified dementia diagnosis, but the share varied within different care settings (61 % in the primary care only, 20 % in the specialist, with no follow-up group and 6 % in the specialist, with follow-up group). Among the newly diagnosed individuals, 47 % were receiving symptomatic anti-dementia treatment. The share was highest in the specialist, with follow-up group (73 %) and lowest in the primary care only group (19 %). The share receiving AChEI respective memantine was the same in the whole population (27 %). There was a difference between groups in the use of AChEIs or the combination of AChEIs and memantine (*p* < 0.01), whereas the observed differences for memantine alone were not statistically significant (*p* = 0.215). In the specialist, no follow-up group, more patients were given memantine compared to AChEI and the pattern was the opposite in the specialist, with follow-up group (Table 2). In all care settings, anti-dementia treatment was most common for patients diagnosed with Alzheimer's Disease, being 52 % in primary care only, 75 % in specialist, no follow-up and 88 % in specialist, with follow-up (**supplement material Figure 1**). The share that was receiving anti-dementia treatment was also highest in the specialist, with follow-up group regardless of diagnosis Table 2.

**Table 2:** Diagnosis and treatment during the first 18 months for newly diagnosed dementia patients, 1st of January 2018 – and 30th of June 2020, stratified by management pathway.

	Care management pathway				
	All	Primary care only	Specialist, no follow-up	Specialist, with follow-up	p-value
n	9784	2492	4671	2621	
Diagnose given, % (95 % CI)					
Alzheimer's	48 (47–49)	16 (15–18)	51 (50–52)	71 (69–73)	<0.01
Unspecified dementia	27 (26–28)	61 (59–63)	20 (19–21)	6 (6–7)	<0.01
Vascular dementia	20 (19–21)	20 (18–21)	23 (22–24)	14 (13–15)	<0.01
Other specified dementia	6 (5–6)	3 (2–4)	6 (5–6)	9 (8–10)	<0.01
Drug treatment, % (95 % CI)					
AChEI or Memantine	47 (46–48)	19 (18–21)	47 (45–48)	73 (71–74)	<0.01
AChEI	27 (26–27)	9 (8–10)	24 (23–25)	48 (46–50)	<0.01
Memantine	27 (26–28)	10 (9–11)	27 (26–28)	44 (42–46)	0.215

**Notes:** Primary care only, a patient only has recorded dementia diagnoses in primary care; Specialist, no follow-up, a patient has a recorded dementia diagnosis in specialist care, but was not followed up in specialist care; Specialist, with follow-up, a patient has a recorded dementia diagnosis in specialist care and was followed up in specialist care**;** the p-value states if there are significant differences between primary care only, specialist, no follow-up and Specialist, with follow-up .

**Abbreviations:** CI, Confidence interval; AChEI, acetylcholinesterase inhibitors.

Most patients were between 80 and 89 years old when they get their first dementia diagnosis. Overall, 7 % of incident cases were diagnosed between the ages of 60 and 69, 31 % between 70 and 79, 45 % between 80 and 89, and 17 % at age 90 or older. The age distribution of those that were followed up in specialist care differed from the those followed up in primary care only. The diagnosis given differed between age groups and care pathways. Alzheimer's was the most frequent diagnosis in care pathway that involves specialist care regardless of age. In total 4241 were diagnosed with Alzheimer's of the 7292 who were diagnosed in a care the pathways including specialist care. However, in the primary care only group unspecified dementia was the most common diagnosis regardless of age. In total 1513 were given unspecified dementia out of the 2492 who were diagnosed in primary care only. The diagnosis of other specified dementia was more common in the youngest age group in all care pathways Fig. 1.

**Fig. 2:**
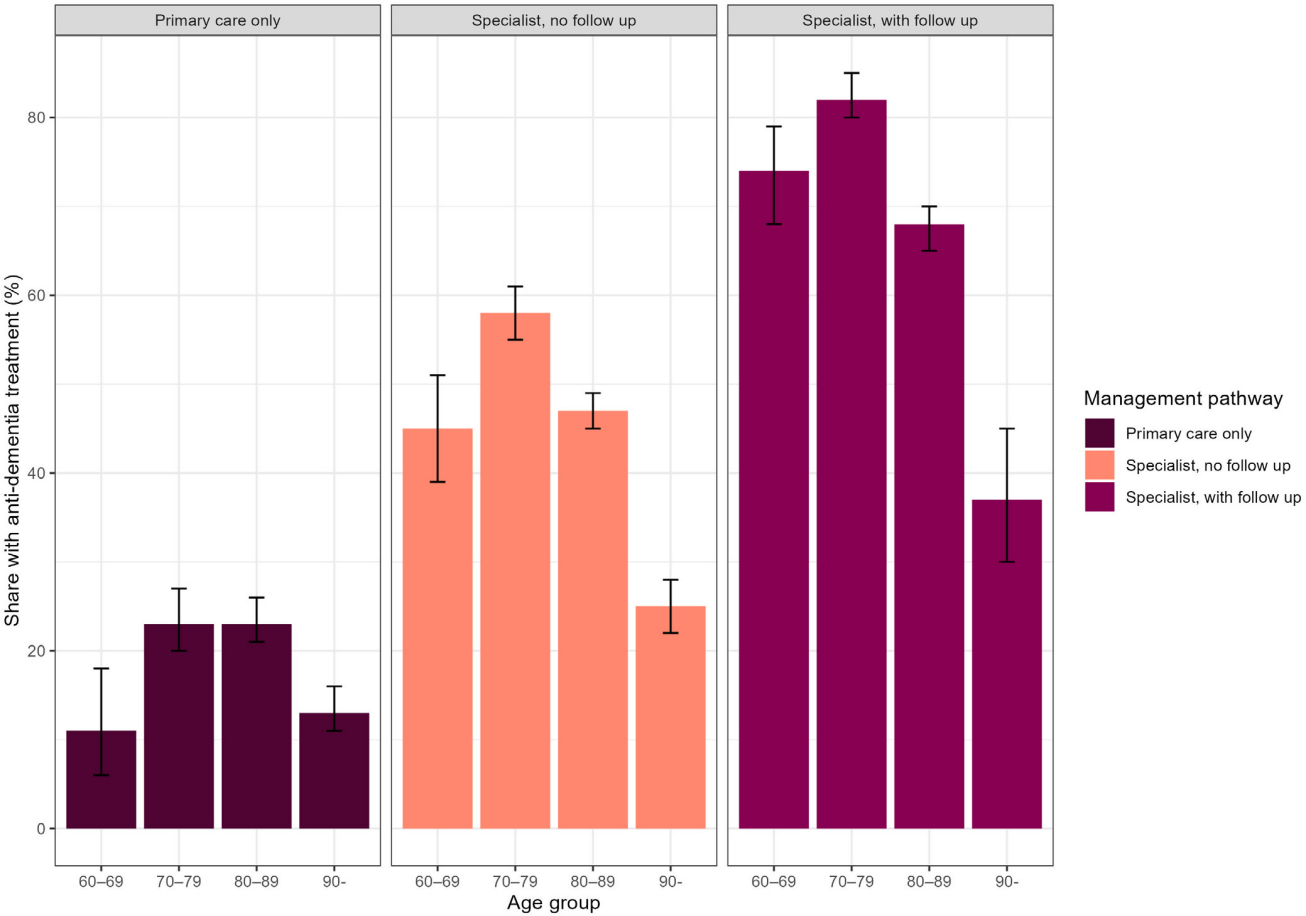
Proportion of newly diagnosed dementia patients receiving anti-dementia drug treatment within 18 months, 1st of January 2018 – and 30th of June 2020, stratified by management pathway and age group, with 95 % confidence intervals. Notes: Primary care only, a patient only has recorded dementia diagnoses in primary care; Specialist, no follow-up, a patient has a recorded dementia diagnosis in specialist care but was not followed up in specialist care; Specialist, with follow-up, a patient has a recorded dementia diagnosis in specialist care and was followed up in specialist care.

In all care pathways, a similar pattern in the share given anti-dementia in different age groups were found. The share given drug treatment increases between the 60–69 and 70–79 age groups and then declined by age. In the age group 60–69 years 11 % in primary care only, 45 % in specialist, no follow up and 74 % in specialist, with follow-up were given anti-dementia treatment. Among patients aged 90 and above, the corresponding proportions were 13 %, 25 %, and 37 %. It was more common to get drug treatment in care pathways that involve specialist care in all age groups Fig. 2.

## 4 Discussion

We analysed a comprehensive sample of all newly diagnosed dementia cases in the Stockholm Region, Sweden, incorporating data on diagnosis and treatment from both primary and specialist care. Studies that include complete information from primary care are rare, making this research a valuable source of new insights that could influence policy decisions.

In our study, 48 % of the dementia cases were diagnosed with Alzheimer's disease. This is lower than other studies reporting the share to be 60 to 80 % [23]. One possible explanation is that our study has a more complete coverage compared to studies recruiting from memory clinics, and thereby include more subjects who have been diagnosed with unspecified dementia. Should these cases undergo further diagnostic evaluation, many might ultimately be diagnosed with Alzheimer's disease. This is demonstrated in a study by Drabo et al., where the diagnosis was revised for 76 % of patients who had initially received an unspecified dementia or non-AD diagnosis [24].

Our results showed that 47 % of all diagnosed cases and 78 % of those diagnosed with Alzheimer's disease were prescribed anti-dementia treatment. This is in line with other studies. For example, an American study reported that 49 % of patients with incident dementia were given anti-dementia treatment [25] and one Korean study reported that 82 % of Alzheimer's disease were given anti-dementia treatment [26]. We observed that the share given treatment varied between care settings. This could be attributed to the fact that patients diagnosed solely in primary care may have more severe dementia at the time of diagnosis and, therefore, may not benefit from treatment to the same extent. This is supported by our finding that this group is older and have more resource utilisation prior diagnosis compared with patients from other care pathways.

For all newly diagnosed individuals, the average care cost per patient one-year prior diagnosis in Stockholm was 321,900 SEK. This is higher than the predicted costs reported in Zilling et al. that predicted formal care costs in Region Skåne, Sweden [27] of about 174,000 SEK. The largest differences compared to our study is the cost of institutionalisation where we observed higher levels of institutionalisation prior diagnosis in Stockholm.

The proportion of patients that are diagnosed and followed up partly or fully in specialist care in this study is 75 %. We expect that this share could be high when compared to other healthcare systems since Region Stockholm has a high availability of memory clinics. Indeed, one study based on Medicare beneficiaries in the US reported that only 15 % were diagnosed by a dementia specialist and that only 22 % a had visit with a dementia specialists within the first year after diagnosis [24].

Our results show that patients with the highest resource utilisations and care costs are the ones that are managed in primary care only. They also to a greater extent do not receive anti-dementia drug treatment. Previous studies have reported average patient care cost by severity stage in dementia to be about 210,000 – 290,000 SEK in mild dementia, 380,000 – 480,000 in moderate and 590,000 – 800,000 SEK in severe ([3,4]). In our study the average annual care cost of patients managed in primary care only was 554,000 SEK which corresponds to the cost of patients with moderate to severe dementia. This suggests that a dementia diagnosis may be part of the process of admitting patients to a care home, which could explain why these patients are not referred to specialist care. The average annual care cost of patients diagnosed and followed up in specialist care was 156,000 SEK which is below the cost of patients with mild dementia. The average care cost of patients diagnosed in specialist care but had no follow-up in specialist care was 291,000 SEK, which corresponds to the cost of patients with mild dementia.

The resource utilisation patterns between groups also differed. In the specialist care groups, both with and without follow-up, outpatient care was more common, whereas inpatient care was more common in the primary care-only group. The lower use of outpatient care in the primary care group could be due to a higher degree of institutionalisation and the use of home care. The higher use of social care in the primary care group may be partly attributed to age, as shown in Aye et al., but is also likely related to higher disease severity [3]. Our study showed that most individuals were referred to specialist care. One reason could be to determine disease pathology before symptomatic drug treatment initiation. Previous studies have shown that clinical diagnosis of Alzheimer's from primary care is unreliable [28]. We can also see that share of anti-dementia drug treatment was higher in the specialist care groups, regardless of age. The cost for an assessment is estimated to be about 7000 SEK in primary care and 12,000 SEK in specialist care (2010 price level) [29]. Since the diagnostic process is more costly in specialist care and there could be a need to free up resources in specialist care to manage new treatments [30], an increase in dementia diagnoses and initiation of symptomatic drug treatment in primary care will be necessary, particularly for patients who will not be eligible for new disease modifying treatments. In this context, diagnostics using new blood-based biomarkers could play a crucial role.

### 4.1 Limitations

This study covers a full sample of patients identified with dementia within the Stockholm region. However, it is limited to documented cases and recorded diagnoses. Consequently, aspects of the diagnostic procedure that are not recorded with a dementia diagnosis are not captured. This includes information on which caregivers or care levels that have referred a patient to specialist care. We have limited the follow-up period to 18 months. Hence, any referrals or drug treatment given after the follow-up period will not be included in the analysis. During three months between March and June 2020 the memory clinics in Region Stockholm were closed due to COVID. This will affect referral patterns during that period and could have an impact on number of incident diagnosis. Additionally, we do not have information on disease severity and cognitive impairment, which could explain differences in care needs. All diagnoses are clinical, and we don't have biomarker data to validate. Hence misdiagnosis will be present in the data. Although the Stockholm region represents an integrated healthcare system serving all residents, the settings and services offered may not accurately reflect those of other healthcare systems. In rural areas where memory clinics are less available as compared to Stockholm, individuals with dementia may use primary care for diagnosis and treatment to a larger extent.

### 4.2 Conclusion

In conclusion, our study enhances the understanding of the dementia management pathway, demonstrating that most patients, at least in the Stockholm region, receive their diagnosis in specialist care, particularly those diagnosed at younger ages. These patients utilised less medical and home care prior to diagnosis. Although the majority of individuals with dementia were diagnosed in specialist care, most were not followed up there, highlighting the crucial role of primary care in managing patients after diagnosis. The high proportion of patients diagnosed in specialist care suggests that a significant share of those eligible for new disease-modifying treatments are already visiting a specialist setting which is the first step of identifying patient to treat. However, with the expected increase in patient influx once new treatments become available, the burden on specialist care will rise. As a result, the role of primary care as a gatekeeper will become increasingly important. Blood-based biomarkers could play an important role in improving diagnostic accuracy in primary care, thereby reducing the need for specialist referrals and a basis for selecting outpatients that could benefit of new treatments methods.

An inverse relationship was observed between resource utilisations prior to diagnosis and the level of specialist management: patients with higher prior resource utilisation were more often managed exclusively in primary care. Older patients were more likely to receive a non-specific dementia diagnosis and were less likely to be prescribed symptomatic anti-dementia drug treatment. This suggests that primary care plays an important role in diagnosing dementia among very old individuals with complex and high-demand needs.

Further research from diverse healthcare systems, including primary care, is needed to compare outcomes and better understand the role of primary care in diagnosing and initiating treatment, particularly in relation to the availability of specialist care and implementation of new treatments and diagnostics.

## CRediT authorship contribution statement

**Emil Aho:** Writing – review & editing, Writing – original draft, Methodology, Formal analysis, Conceptualization. **Dorota Religa:** Writing – review & editing. **Mozhu Ding:** Writing – review & editing. **Bengt Winblad:** Writing – review & editing. **Linus Jönsson:** Writing – review & editing, Conceptualization. **Karin Modig:** Writing – review & editing, Data curation, Conceptualization.

## Declaration of competing interest

The authors declare the following financial interests/personal relationships which may be considered as potential competing interests:

Emil Aho reports financial support was provided by Sweden's Innovation Agency. Bengt Winblad reports a relationship with Artery Therapeutics Inc, AlzeCure, Alzinova that includes: consulting or advisory. Linus onsson has patent with royalties paid to Resource Utilisation in Dementia (RUD) instrument. Bengt Winblad has patent with royalties paid to Resource Utilisation in Dementia (RUD) instrument. Linus Jönsson has received consulting fees from H. Lundbeck A/S, Eli Lilly Inc and Novo Nordisk A/S If there are other authors, they declare that they have no known competing financial interests or personal relationships that could have appeared to influence the work reported in this paper.

## Appendix Supplementary materials

Image, application 1
