# Supplementary material for: Patient management pathways in dementia – Resource utilisation, diagnosis and drug treatment in the Stockholm region, Sweden
Source: J Prev Alzheimers Dis. 2025 Mar 17;12(6):100132. doi: 10.1016/j.tjpad.2025.100132 (PMC12434265; doi:10.1016/j.tjpad.2025.100132)
Supplement: Supplementary file 1 [file mmc1.docx]

**Table 1 Unit costs**

|  | **Cost SEK (2022 price level)** |
| --- | --- |
| Primary care (cost per visit) | 1,832 |
| Home help services (cost per hour) | 571 |
| Day time activities (cost per month) | 9,792 |
| Short term care (cost per day) | 3,232 |
| Institutionalisation (cost per month) | 90,238 |


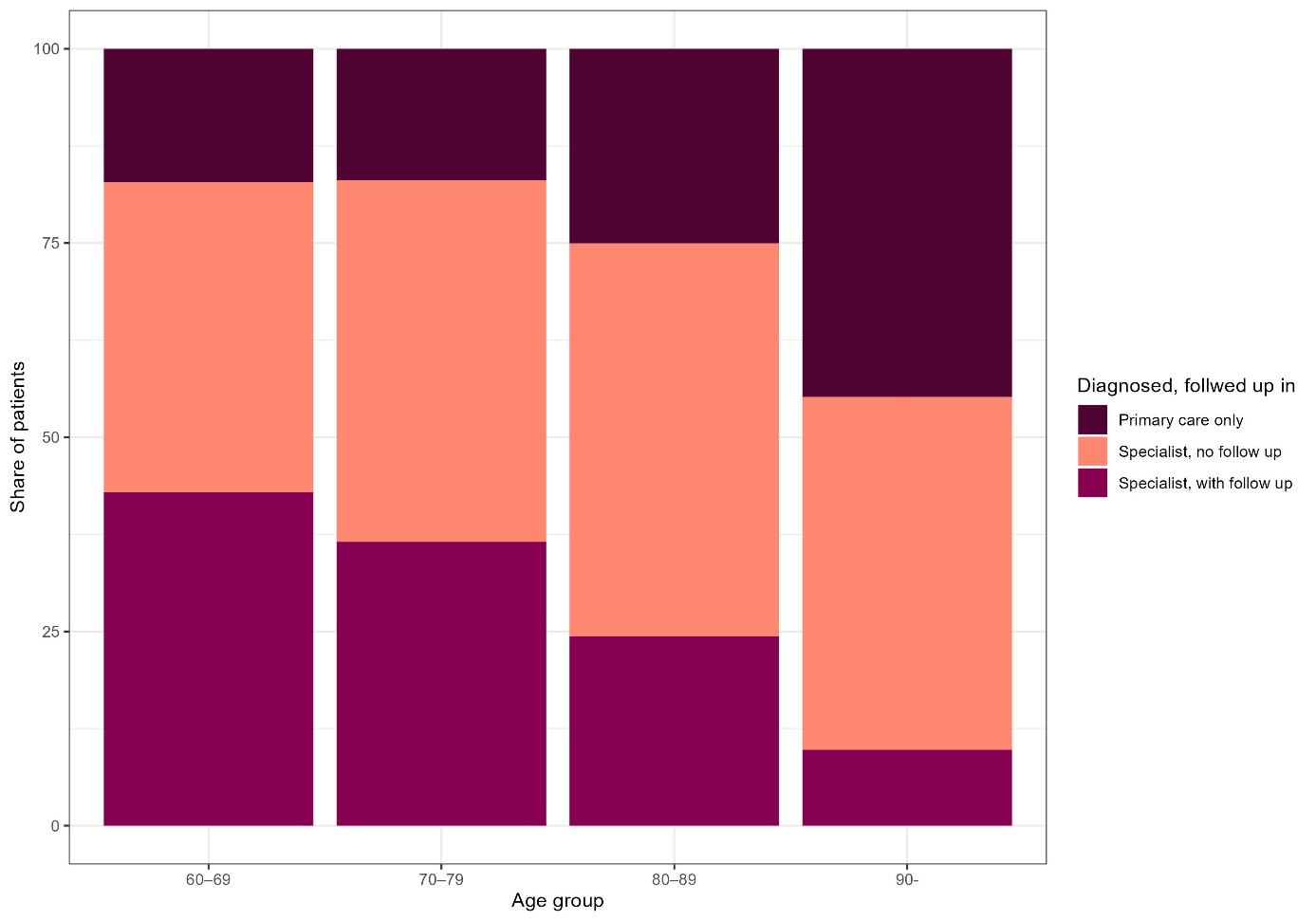


**Figure 1 Share of patients diagnosed and followed up in different care settings for newly diagnosed dementia patients, 1^st^ of January 2018 – and 30^th^ of June 2020, stratified age group.**

**Notes:** Primary care only, a patient only has recorded dementia diagnoses in primary care; Specialist, no follow-up, a patient has a recorded dementia diagnosis in specialist care but was not followed up in specialist care; Specialist, with follow-up, a patient have a recorded dementia diagnosis in specialist care and was followed up in specialist care**.**

**
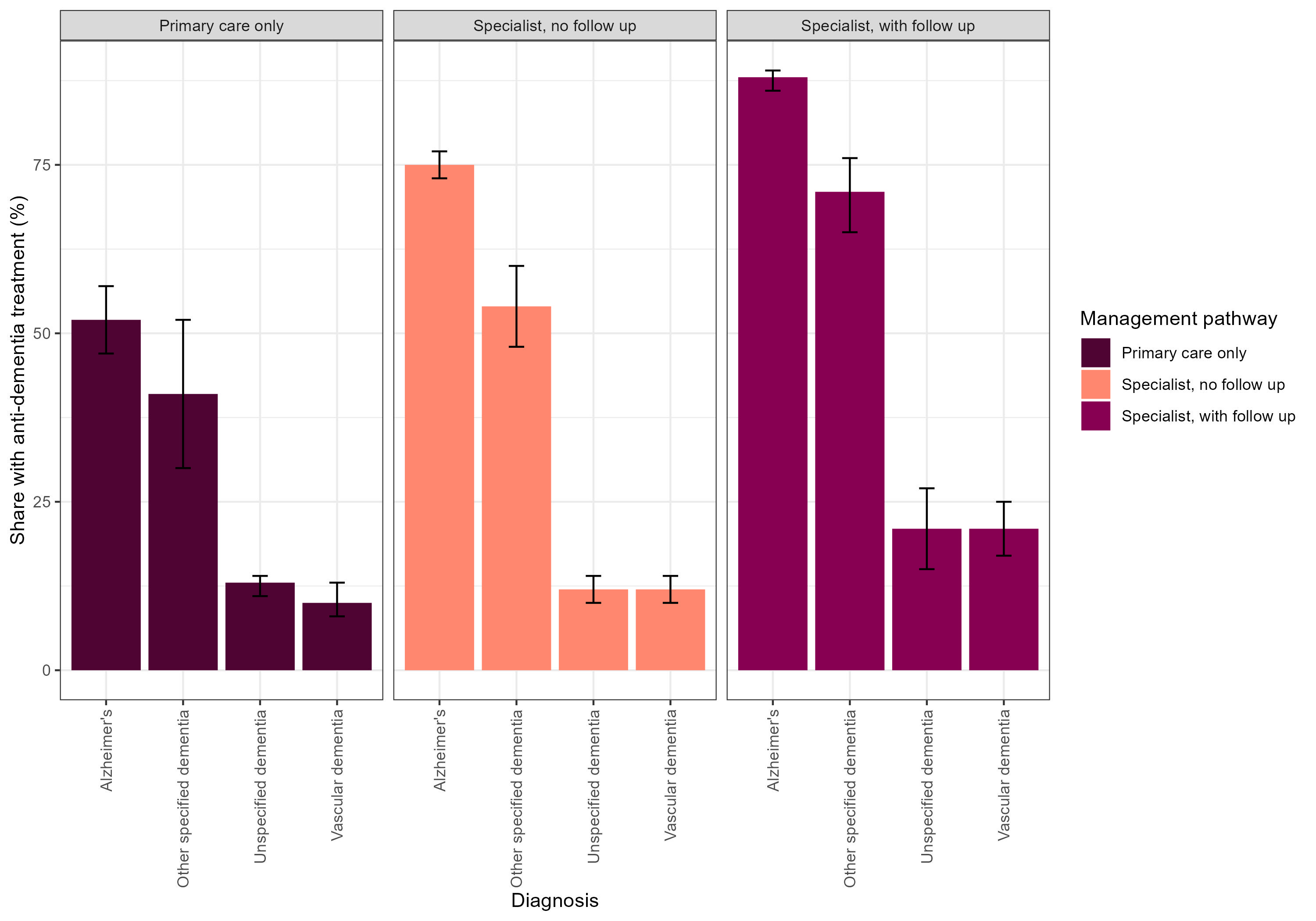
**

**Figure 2 Proportion of newly diagnosed dementia patients receiving anti-dementia drug treatment within 18 months, 1^st^ of January 2018 – and 30^th^ of June 2020, stratified by management pathway and diagnosis, with 95% confidence intervals.**

**Notes:** Primary care only, a patient only has recorded dementia diagnoses in primary care; Specialist, no follow-up, a patient has a recorded dementia diagnosis in specialist care but was not followed up in specialist care; Specialist, with follow-up, a patient have a recorded dementia diagnosis in specialist care and was followed up in specialist care**.**
